# Characterization of Early-Stage Alcoholic Liver Disease with Hyperhomocysteinemia and Gut Dysfunction and Associated Immune Response in Alcohol Use Disorder Patients

**DOI:** 10.3390/biomedicines9010007

**Published:** 2020-12-24

**Authors:** Vatsalya Vatsalya, Khushboo S. Gala, Ammar Z. Hassan, Jane Frimodig, Maiying Kong, Nachiketa Sinha, Melanie L. Schwandt

**Affiliations:** 1 Department of Medicine, University of Louisville, Louisville, KY 40202, USA; khushboo.gala@louisville.edu (K.S.G.); ammarzahidhassan@gmail.com (A.Z.H.); jane.frimodig@louisville.edu (J.F.); 2 Department of Biostatistics and Bioinformatics, University of Louisville, Louisville, KY 40202, USA; maiying.kong@louisville.edu; 3 Department of Psychiatry, Dalhousie University, Halifax, Nova Scotia, NS B3H 4R2, Canada; nachiketa.sinha@gmail.com; 4 National Institute on Alcohol Abuse and Alcoholism, Bethesda, MD 20892, USA; melanie.schwandt@nih.gov

**Keywords:** ALD, AUD, heavy drinking markers, hyperhomocysteinemia, TLFB, withdrawal

## Abstract

Heavy alcohol consumption can cause hyperhomocysteinemia, which could be consequential in the proinflammatory response and worsening of the neurobehavioral domains of alcohol use disorder (AUD), such as alcohol withdrawal. We examined the role of heavy drinking, hyperhomocysteinemia, gut dysfunction and inflammation in early-stage alcoholic liver disease (ALD) in AUD patients. A total of 110 AUD patients without clinical manifestations of liver injury were grouped by the serum homocysteine levels (SHL): normal ≤ 13 µmol/L (Group 1 (Gr.1); *n* = 80), and elevated > 13 µmol/L (Group 2 (Gr.2), *n* = 30). A comprehensive metabolic panel, SHL, a nutritional assessment, and drinking history assessed by the timeline followback questionnaire were evaluated. A subset analysis was performed on 47 subjects (Gr.1 *n* = 27; Gr.2 *n* = 20) for additional measures: Clinical Institute Withdrawal Assessment for Alcohol (CIWA) score, plasma cytokines (interleukin-1β (IL-1β)), gut dysfunction markers (lipopolysaccharide (LPS), and LPS-binding protein (LBP)); 27% of the AUD patients exhibited hyperhomocysteinemia. SHL was significantly associated (*p* = 0.034) with heavy drinking days (HDD90). Subset analyses showed that the withdrawal ratings were both clinically and statistically (*p* = 0.033) elevated and significantly associated with hyperhomocysteinemia (*p* = 0.016) in Gr.2. LBP, IL1-β, SHL, and HDD90 showed significant cumulative effects (adjusted R^2^ = 0.627) on withdrawal ratings in Gr.2 subset. Alanine aminotransferase (ALT) and aspartate aminotransferase (AST) were significantly higher in all Gr.2 patients; AUROC showed a fair level of true positivity for ALT (0.676), and AST (0.686). Il1-β, LBP, SHL, and HDD90 showed significant cumulative effects (adjusted R^2^ = 0.554) on the elevated ALT in Gr.2 subset as well. The gut-brain derived proinflammatory response, patterns of heavy drinking, and hyperhomocysteinemia were closely associated with clinically elevated alcohol withdrawal and elevated liver injury. Hyperhomocysteinemia could have a potential phenotypic marker response indicative of early-stage ALD along with AUD.

## 1. Introduction

Alcohol use disorder (AUD) is a multi-factorial disease [1] that involves various neurobehavioral changes and dysregulation of key metabolic mechanisms that involve nutritional alterations [2]. Elevated serum homocysteine levels (SHL) have been observed both in chronic alcohol use and alcohol-associated organ injury [3,4,5,6]. In acute alcohol withdrawal, interaction of SHL and agonism at the *N*-methyl-d-aspartate receptor may partly mediate alcohol-associated withdrawal symptomatology [7]. Chronic alcohol use has been known to interfere with methionine metabolism and leads to increased levels of homocysteine and S-adenosylhomocysteine (SAH), as well as decreased levels of hepatic S-adenosylmethionine (SAMe) [3]. Two pathways, the methionine cycle and transsulfuration, account for most of the methionine metabolism in mammals; among these, trans-sulfuration occurs primarily in the liver (hepatocytes, apart from the kidney, pancreas, and small intestine) that leads to the catabolism of homocysteine [8]. Hyperhomocysteinemia in alcoholics is also exacerbated by a lack of dietary elements that serve as cofactors (i.e., Vitamin B12, B6, and folate) for hepatic methionine metabolism [9]. Elevated homocysteine levels have been observed with the consumption of every spirit and wine in moderate drinkers [10]. The Franconian Alcoholism Research Studies (FARS) reported elevated homocysteine levels in 144 non-abstinent heavy drinkers; [11] however, patterns of drinking and recent drinking assessments were not part of this study.

Alcoholic liver disease (ALD) is a pathological consequence (organ-system complication) of AUD [12,13]. ALD is a spectrum of clinical conditions that ranges from hepatic steatosis to advanced forms such as steatohepatitis/cirrhosis [14]. The underlying mechanisms through which heavy and chronic alcohol consumption lead to liver disease are complex and involve multiple factors [15]. Nutrition seems to play a major role in liver health as well as in the treatment and prognosis of ALD. One of the pathways involved in liver injury is altered methionine metabolism with hyperhomocysteinemia [14,16]. This milieu of dysregulated methionine metabolism leads to the generation of proinflammatory cytokines and increased oxidative stress that can initiate and foster immune-mediated liver damage and disease progression observed in alcoholic liver disease [17,18,19]. For example, chronic alcohol feeding in rats caused increased accumulation of homocysteine in adipocytes, with corresponding alterations in adiponectin production and hepatocyte damage [20].

There are gaps in the understanding of the drinking level and rate responsible for changes in the homocysteine response in heavy drinkers diagnosed with AUD. Thus, we hypothesized that homocysteine levels will correspond significantly with the drinking history and markers in alcohol use disorder (AUD) patients, and with alcohol withdrawal as a neurobehavioral component of the gut-brain proinflammatory axis. Although there is much information on the role and effect of serum homocysteine in alcoholic liver disease, no clinical studies have determined the relationship of homocysteine, markers of heavy drinking, proinflammatory gut-liver axis, and liver injury measures all together. We also identified the role of hyperhomocysteinemia in early-stage ALD. Changes in homocysteine with alcohol intake and underlying sex differences are also not well studied in heavy drinking individuals. We examined the changes in the levels of homocysteine and their association with drinking markers and liver injury in males and females independently.

## 2. Materials and Methods

### 2.1. Patient Recruitment

This study was approved by the Institutional Review Board of the National Institute on Alcohol Abuse and Alcoholism at the National Institutes of Health, Bethesda MD, under the screening protocol 98-AA-0009 (November 3, 1999 yr.–ongoing). The study was indexed at the National Clinical Trial website (www.clinicaltrials.gov: NCT00001673). In total, 110 male and female individuals aged 21–65 years were included in this single time-point clinical observational study (Table 1). Written informed consent was obtained from all participants. All the assessments included in this study were collected at the time of screening for admission in this investigation after the completion of the consenting process to participate in the study and to provide clinical information and bodily samples. Patients were diagnosed with alcohol dependence (AD) according to the Diagnostic and Statistical Manual of Mental Disorders-IV (DSM-IV), based on the alcohol dependence module of the SCID I-interview. More detailed eligibility criteria and study design concepts are available in our previous publications [15,21]. Patients with an outlier level of ALT (more than 400 IU/L) and diagnosis of alcoholic hepatitis/cirrhosis or history of enzymatic deficiencies of methionine pathways were excluded from this investigation.

### 2.2. Demographics, Drinking, and Laboratory Assessments

Demographics (Age, Sex, Body Mass Index (BMI)) and recent drinking history were collected and used in the analysis as factors and covariates. Recent drinking measures were collected from the Time-line Follow-Back questionnaire [22] (which included Total Drinks past 90 Days (TD90), Number of Drinking Days past 90 Days (NDD90), Number of Non-Drinking Days past 90 Days (NNDD90), Average Drinking Days (AvgDD90), and Heavy Drinking Days past 90 Days (HDD90)). Lifetime drinking history (LTDH) was another alcohol inventory questionnaire that was used in this study [23]. Both these questionnaires are well established and validated to be better than the laboratory markers [24,25]. Data from these were used as drinking markers. We used “Controlling Nutritional Status Test” (CONUT) data to establish nutritional status [26]. CONtrolling NUTritional status [CONUT or CAL score] was derived from the categorical levels of serum total serum cholesterol (mg/dL), serum albumin (g/dL), and total lymphocyte count (/mL of blood). This was performed to estimate that the patients did not have any nutritional deficiency that could alter the homocysteine levels. We did not find any statistical or numerical differences between the groups (Data not reported). The Clinical Institute Withdrawal Assessment for Alcohol scale (CIWA-Ar) was collected to evaluate the alcohol withdrawal in a sub-set of 47 AUD patients (27 with normal homocysteine levels and 20 with hyperhomocysteinemia) [27]. We used ≥10 as our eligibility criteria for a diagnosis based on mild and above severity of withdrawal for identifying withdrawal (http://www.regionstrauma.org/blogs/ciwa.pdf).

On the day of evaluation, blood samples were drawn for a serum chemistry panel (Table 1). Alanine aminotransaminase (ALT) levels were used as the reference measure to assess liver injury (Medline Plus-National Institutes of Health, 2014). ALT values of 40 IU/L represented the upper limit of normal, while values >40 indicated mild liver injury as per the guideline until 2014 [15]. This cut-off has been used in several previous publications for early ALD [15,21]. Aspartate aminotransferase (AST) was considered clinically significant at >34 IU/L. Serum chemistry was used to determine potential homocysteine levels. We used the homocysteine level as a primary discriminating factor for the association with the liver injury in this study. Thus, we grouped patients with normal serum homocysteine level (0–13 µmol/L) as Gr.1 and high (≥14 umol/L) as Gr.2. All laboratory measures were analyzed with the guideline set-up at the Medline Plus (till 2014) and the Department of Laboratory Medicine. All laboratory tests were performed by the Department of Laboratory Medicine at the National Institutes of Health.

### 2.3. Laboratory Analysis

On a small sub-set of AUD patients (*n* = 47), cytokines and gut permeability markers were evaluated. Plasma pro-inflammatory cytokine interleukin 1β was determined by multianalyte chemiluminescent detection using Multiplex kits (Millipore, Billerica, MA, USA) on the Luminex (Luminex, Austin, TX, USA) platform based on the manufacturers’ instructions. Plasma lipopolysaccharide (LPS) and LBP (LPS binding protein) levels were analyzed using the Kinetic Chromogenic Limulus Amoebocyte Lysate Assay (Lonza, Walkersville, MD, USA) based on the manufacturer’s instructions.

### 2.4. Statistical Analysis

One-way ANOVA was used to evaluate differences in the demographic characteristics, drinking history measures, and liver injury markers. Univariate analysis of covariance (ANCOVA) was used to evaluate differences in the liver injury markers between the two homocysteine level groups (overall) and by sex within the two homocysteine groups as a factor. The gut-derived proinflammatory response was evaluated for alcohol withdrawal and alcoholic liver disease. Regression analysis was used to characterize the association of the homocysteine level, liver injury, and involvement of drinking history measures (as secondary independent variables) by sex differences. Linear regression analysis with either single independent variable or multiple independent variables (identification of mediating role) was used to associate homocysteine levels and liver injury in the context of drinking markers. Receiver operating characteristic (ROC) curves were further constructed to examine the sensitivity and specificity of the ALT and AST levels between Gr.1 and Gr.2. This investigation had a large number of study participants; thus, the sample size was more than adequate to address and support the statistical analyses. SPSS 26.0 (IBM, Chicago, IL, USA) and Microsoft office 365 Excel (MS Corp, Redmond, WA, USA) were used for statistical analysis and data computation. Statistical significance was established at *p* ≤ 0.05. Data are expressed as the Mean ± SD (standard deviation) in the tables.

## 3. Results

### 3.1. Patient Demographics, Drinking Markers, and Liver Injury Assessment

In total, 110 AD patients were enrolled in the study, 73 males and 37 females. Among these, 30 patients (Gr.2) had clinically significant elevated serum homocysteine levels, thus around 27% of AUD patients exhibited clinically significant hyperhomocysteinemia (Table 1). Mean BMI values were not significantly different between the two groups; however, age was statistically significant but ranged in the middle age criteria only (Table 1). Distributions of males and females in each of the two groups were similar. Patients were not clinically malnourished, and the nutrition status as assessed by the CONUT (Controlling nutritional status) test was not different between the two groups. Since demographic measures and CONUT did not show any relevant difference, we did not incorporate these measures as covariates in the analyses unless clinically indicated. We did not find any significant difference in the drinking history markers between the two groups.

### 3.2. Association of Serum Homocysteine and Drinking Markers, and Alcohol Withdrawal

**Figure 1:**
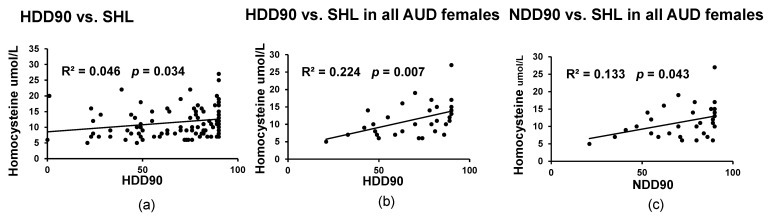
Relationship between serum homocysteine level (SHL) and drinking markers. (**a**) Significant positive association between SHL and heavy drinking days past 90 days (HDD90). (**b**) Significant positive association between SHL and HDD90 in all AUD females. (**c**) Significant positive association between SHL and number of drinking days past 90 days (NDD90) in all AUD females. Statistical significance was set at *p* ≤ 0.05. R^2^ denotes the effect of the relationship as an outcome of univariate linear regression.

SHL was significantly associated only with the HDD90 values (with a low effect) in all the study patients (Figure 1a); this association was largely driven by the significant positive linear association in all AUD female patients (Figure 1b). SHL and NDD90 values were closely associated in a linear positive manner for AUD females only (Figure 1c). No such associations were found in male patients. This is because HDD90 was much higher in Gr.2 females (77.7 vs. Gr.1 females at 65 (See Table 1), a difference of 12 units). Due to this, SHL was likely more associated with females. However, between the males of both the groups, this difference was in fact the opposite (Gr. 2 males had 3 units of HDD90 less than Gr.1 males). Gr.2 SHL was not associated with any of the drinking markers, largely due to the fact that liver injury was not clinical, and at an early stage, likely only the liver function was affected. On the contrary, in the Gr.1 AUD patients with normal homocysteine, SHL was significantly associated with HDD90 (*p* = 0.001) and NDD90 (*p* = 0.003), implying that drinking markers lose predictability for SHL when SHL is clinically high (data not plotted).

**Figure 2:**
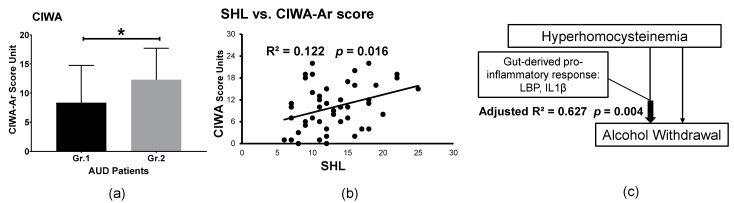
Role of the serum homocysteine level (SHL) in alcohol withdrawal in a sub-set of *n* = 47 subjects. (**a**) Significant elevation in CIWA-Ar scores in Gr.2 AUD subjects with elevated SHL. Data are presented in Figure 2a as the mean ± standard deviation. R^2^ denotes the effect of the relationship as an outcome of univariate linear regression (Figure 2b) or as adjusted R^2^ with multivariable analysis (Figure 2c). Statistical significance was set at * *p* ≤ 0.05 (Figure 2a). (**b**) Significant positive association of SHL and the CIWA-Ar score. (**c**) Role of hyperhomocysteinemia; heavy drinking marker, HDD90; and gut-derived proinflammatory response consequential in higher withdrawal ratings in Gr. 2 AUD patients.

We evaluated alcohol withdrawal, one of the neurobehavioral domains of alcohol use disorder, in a sub-set analysis (*n* = 47) between the two groups (from Gr.1, *n* = 27; and from Gr.2, *n* = 20), for which these data were collected. CIWA was clinically not significant in Gr. 1(M ± SD: 8.1 ± 6.26) whereas in Gr.2, it was clinically significant (M ± SD: 12.3 ± 5.86) (Figure 2a). This elevation in CIWA in Gr.2 showed a statistical difference in the context of the pro-inflammatory response (covaried with interleukin 1β (IL1β)). SHL was significantly associated with alcohol withdrawal (Figure 2b) in Gr.2. A pathway analysis model showed that hyperhomocysteinemia and HDD90 along with LBP and IL1β showed a significant cumulative effect on alcohol withdrawal in Gr. 2 (Figure 2c) that was not evident in Gr.1.

### 3.3. Evaluation of Liver Injury Markers and the Role of Homocysteine

**Figure 3:**
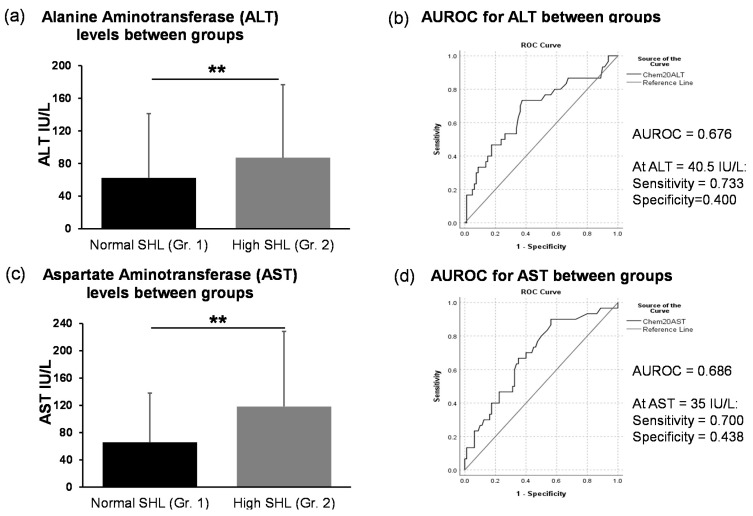
Difference in alanine aminotransferase (ALT) and aspartate aminotransferase (AST) levels between the alcohol use disorder (AUD) patients with normal homocysteine levels (Gr. 1) and with high homocysteine levels (Gr. 2). (**a**): Significantly elevated ALT levels in Gr. 2 compared to Gr.1. (**b**): Area under the curve for receiver operating characteristic in the ALT levels between Gr. 1 and Gr. 2. (**c**): Significantly elevated AST levels in Gr. 2 compared to Gr.1. (**d**): Area under the curve for receiver operating characteristic in the AST levels between Gr. 1 and Gr. 2. Statistical significance was set at ** *p* ≤ 0.05 (Noted in Figure 3a,c). Data are presented as the mean ± standard deviation. AUROC is presented for Gr.2 in comparison to Gr. 1 with the sensitivity and specificity responses at the clinical margins of ALT and AST.

Liver injury markers ALT and AST were both clinically and statistically significant in the Gr.2 patients, who also exhibited high homocysteine (Table 1, Figure 3). ROC curve analysis showed a fair level of true positivity in the differences in ALT and AST levels between the two groups. These subtle levels can be anticipated with early-stage ALD. SHL was very significantly associated with ALT and AST independently in all the study subjects (*p* ≤ 0.01). When we performed the association analyses within each group, we found that ALT and AST levels were significantly associated with SHL in Gr.1 only, i.e., not in Gr.2 (Data not plotted), suggesting that SHL and liver injury markers show corresponding levels when SHL was in the normal range only. Elevated SHL levels corresponded well with liver injury (Figure 3a,c); however, several pathways can be involved, thus association of SHL and liver injury should be investigated by including other interacting pathological factors (such as gut dysfunction and pro-inflammatory cytokines) apart from the heavy drinking patterns.

AST also showed a significant albeit mild association with HDD90 (*p* = 0.037) and NDD90 (*p* = 0.038) in Gr.2 (data not plotted). In the normal homocysteine group, we did not find any significant association of liver injury marker ALT and drinking measures. The AST:ALT ratio (a robust marker of progression of ALD, for example, alcoholic hepatitis) was in a normal clinical range overall, as well as within each study group. It was not associated with homocysteine levels overall, as well as within each study group, suggesting that the ALD had not yet progressed and remained at an early stage.

**Figure 4:**
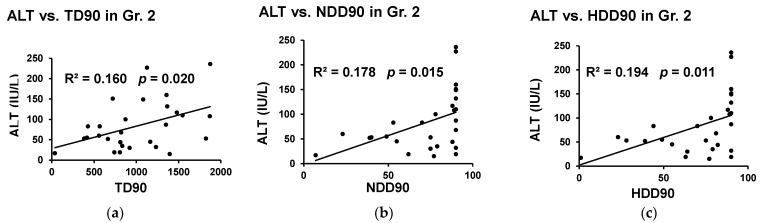
Association of ALT and markers of heavy drinking in the elevated homocysteine group of AUD patients (Gr. 2). (**a**) Significant association of ALT and total drinks past 90 days (TD90). (**b**) Significant association of ALT and NDD90. (**c**) Significant association of ALT and HDD90. Statistical significance was set at *p* ≤ 0.05. R^2^ denotes the effect of the relationship as an outcome of univariate linear regression.

**Figure 5:**
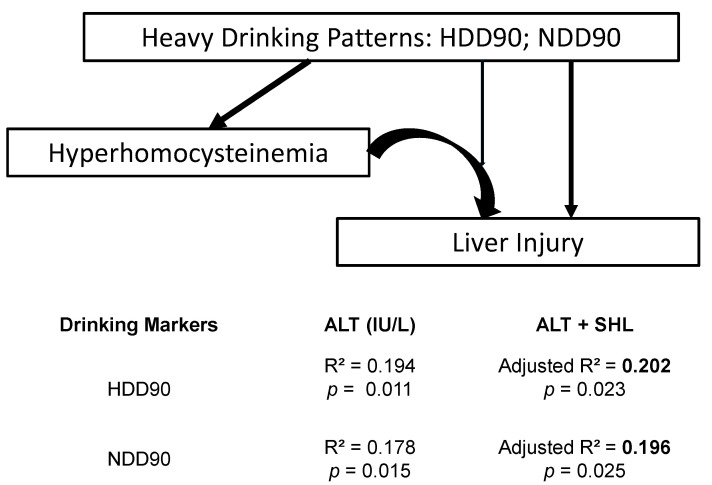
Direct response of the drinking markers on liver injury and the mediating role of the serum homocysteine level (SHL) in AUD patients with high homocysteine levels (Gr. 2). The indirect role of SHL shows higher effects of association together with heavy drinking in the exacerbation of the liver injury. Statistical significance was set at *p* ≤ 0.05. R^2^ denotes univariate effect sizes. Adjusted R^2^ (in Bold font) denotes the effect of the relationship as an outcome of multivariate linear regression with the added variable of SHL.

Gr.2 patients showed a significant association of ALT and several drinking markers independently. TD90 and NDD90 drinking markers were significantly associated with ALT (Figure 4a,b). To note, ALT showed the strongest association with HDD90 (Figure 4c). Importantly, SHL showed mediating effects on the drinking markers in predicting the liver injury marker ALT (Figure 5). HDD90 and NDD90 drinking patterns showed a significant albeit weak association with the liver injury marker ALT (2nd column, Table of Figure 5). The augmented effect sizes of HDD90 and NDD90 on ALT support the role of SHL in exacerbating the effects on the liver injury (3rd column of the table of Figure 5). We further explored the role of the gut-liver axis involved in the proinflammatory response and involvement of hyperhomocysteinemia and heavy drinking on ALT. In the pathological paradigm as illustrated in Figure 5, we found that by including the markers of gut dysfunction and proinflammatory response, (LBP, and IL1β), a significant and robust effect on ALT (adjusted R^2^ = 0.554 at *p* = 0.010) can be ascertained.

## 4. Discussion

Elevated SHL values have been observed in acute alcohol withdrawal as well as alcohol use disorder [7]. Our study showed that specific markers of heavy drinking had a more significant impact on SHL with a propensity in female patients. SHL was significantly associated with the number of heavy drinking days in all AUD patients; however, this was largely driven by the vulnerability in the female patients, which contributed largely to this relationship. In female patients, SHL was also positively associated with NDD90. Thus, both the frequency and intensity of heavy alcohol intake influenced SHL levels in females. Interestingly, studies on non-alcoholic fatty liver disease (NAFLD) have shown that SHL has no relation with NAFLD in women, but positive associations in men [28,29]. It has also been shown that elevated SHL and coronary artery disease have no relation in females [30,31]. This supports that a sex-based association between SHL and alcohol use disorder is uniquely different from other diseases, which has not been previously described. We also found that SHL was associated with drinking markers only when in the normal range and lost significance in the elevated SHL group, which demonstrates that drinking markers lose predictability for SHL with hyperhomocysteinemia. From our data, we can interpret that SHL could be associated with altered liver function when signs of early-stage ALD might or might not be observed. This is due to a direct role of alcohol pharmacology in liver that changes SHL levels, and later SHL could show corresponding changes when liver injury is present.

Elevated SHL values have been associated with increased severity of alcohol withdrawal and alcohol-withdrawal seizures [7,32]. Furthermore, hyperhomocysteinemia in AUD patients can lead to impaired cerebral blood flow and progression of dementia and neurotoxicity [6,33]. There is an upregulation of NMDA receptors in certain brain areas, which is an adaptive consequence of chronic alcohol consumption; homocysteine and its catabolic products serve as neurotransmitters and agonists for these receptors, and thus have been implicated in withdrawal and associated seizures [34,35,36]. Homocysteine is an intermediate product in the metabolism of the amino acids, such as methionine and cysteine. Our analysis also shows significant correlations of CIWA-Ar scores with hyperhomocysteinemia (adversity in one of the neurobehavioral domains of AUD), consistent with previous reports [32]. When we expanded this finding to the gut-brain axis as a novel mechanism, we found a significantly stronger effect of positive interaction of the gut-derived pro-inflammatory response. We developed a pathway analysis model based on our results, which showed that elevated SHL and heavy drinking (as explained by high HDD90) along with the gut permeability alterations (LBP) and proinflammatory response (IL1β, likely produced from the macrophage cells) [37] had a very strong and statistically significant cumulative effect on alcohol withdrawal.

Alcohol itself is implicated in the dysregulation of homocysteine. Chronic alcohol consumption leads to posttranslational modification of specific enzymes in hepatic methionine metabolism, resulting in hyperhomocysteinemia [38,39]. The enzyme methionine synthase is inhibited by alcohol use, decreasing re-methylation of homocysteine [40]. Alternate pathways for remethylating homocysteine, such as betaine homocysteine methyltransferase, are also initially sensitized with observed elevation and an adaptive response that ultimately shows exhaustion with chronic alcohol abuse [41].

We found that markers of liver dysfunction, serum ALT and AST, were both clinically elevated and statistically significant in patients with hyperhomocysteinemia as compared to those with normal homocysteine levels. ALT and AST are good indicators in early-stage liver injury; however, clinical determinants are needed to explain liver injury in advanced ALD (for example, alcoholic cirrhosis and hepatitis) that can show clinical severity (AST:ALT ratio, MELD, Maddrey ABIC etc.). Notably, we found that transaminase levels were significantly associated in patients with normal SHL but not in elevated SHL, suggesting that SHL and liver injury markers show coherence in normal ranges. Once liver injury is observed in AUD patients, SHL, drinking patterns, and other important factors such as gut dysfunction and proinflammation become involved. Specifically, our study showed associations between ALT and drinking markers and elevated SHL in AUD patients that were notably augmented in the context of the gut dysfunction and cytokine response. Importantly, SHL did not show a direct relationship with liver injury, but along with the heavy drinking markers, predicted elevated ALT. This supports the role of SHL in exacerbating liver injury when gut dysfunction and the pro-inflammatory response come into play in early-stage ALD. Elevated homocysteine levels can be found in all stages of ALD, from the developmental stages of ALD to advanced forms such as alcoholic cirrhosis [5,42,43]. The byproducts of methionine metabolism in ALD (marked by the lowered SAMe levels leading to competitive inhibition of most methyltransferases and upregulated demethylated product as homocysteine [44] and its derivatives such as SAH [8]) interact with proinflammatory cytokines (such as TNF-α), subsequently potentiating hepatotoxicity and fatty liver [17].

As with analysis of alcohol withdrawal, we added markers of the gut-liver axis and proinflammatory response to the analysis and found a significant increase in the effect on ALT. Studies on both animal and human models support that chronic alcohol exposure induces gut-derived altered immunomodulatory effects, leading to the activation of proinflammatory cytokine pathways, [45,46] as was also observed in our study. Thus, there was a multifactorial consequence in the liver injury.

Homocysteine causes hepatic injury due to its involvement in several key mechanisms [18]. It activates necrosis factor-κB (NF-κB) and increases the production of proinflammatory cytokines, resulting in inflammatory reactions [17,47]. It also leads to endoplasmic reticulum (ER) stress by causing misfolding of proteins traversing the ER [48]; this mechanism is thought to play an important role in inducing hepatic steatosis, and recent research has implicated this in the pathophysiology of non-alcoholic fatty liver disease [49]. Lastly, it causes oxidative stress by increasing intracellular levels of superoxide anions [50].

There were some limitations in this clinical study. This is a clinical observational study and was conducted as a single timepoint investigation. Thus, we do not have longitudinal data on homocysteine, or on any treatment and changes in ALD and AUD with respect to homocysteine levels. Also, we have not presented outcomes for the role of ethnicity and race, which was not in the scope of this study. Demographic data showed that race was shifted toward patients who reported as Caucasian (*n* = 62), with smaller numbers reporting as Asian (*n* = 2), Latino (*n* = 12), and African-American (*n* = 34). Among the African Americans in our cohort, 27 were males and only 7 were females. This created a statistical problem for analysis, so they were not analyzed as a separate group. Another limitation of this study is that we do not have the dietary history on the intake of SAMe. However, a previous study suggested that daily macro- and micronutrient intake does not seem to be responsible for the sex-specific susceptibility in the development of ALD [18]. Further, none of these patients was overtly malnourished to indicate a variability in nutrition, and both groups (and their sub-groups divided by sex) showed equivalent borderline overweight average BMIs. Individual variability among females was observed and likely there are female-specific factors that might come into play that could only be identified with mechanistic studies. We did not conduct preclinical studies on animal models to identify the precise mechanisms involved.

Our findings support the role of SHL in AUD and its neurobehavioral domain as well as in liver injury and thus ascertain a greater significance of lowering excessive alcohol in individuals who also show elevated serum homocysteine levels. Elevated homocysteine levels have been used as a screening method to identify patients at risk for complications of withdrawal such as seizures, [32] and have also shown adverse effects involved in the pathogenesis of ALD. Our study identified the context of heavy drinking markers, thus characterizing the hyperhomocysteinemia categorically, as well as illustrating the central mechanism of gut dysfunction that was common for the proinflammatory response in AUD and ALD distinctly in the same patient cohort. Hyperhomocysteinemia was identified as a translational marker of AUD and ALD.

## Figures and Tables

**Table 1:** Demographic, drinking history assessment, nutritional, liver injury, and gut-barrier and derived markers in AUD patients.

Measures	Group 1 (Normal Homocysteine, GR.1)				Group 2 (Elevated Homocysteine, GR.2)				*p*-Value (Gr. 1 & Gr. 2)
	Males (*n* = 53; 66%)	Females (*n* = 27; 34%)	Within gr. sex-diff. *p*-Value	Total (*n* = 80; 71%)	Males (*n* = 19; 61%)	Females (*n* = 11; 39%)	Within gr. sex-diff. *p*-Value	Total (*n* = 30; 27%)	
Age (years)	38.85 ± 10.9	42.84 ± 9.9	NS	40.2 ± 10.7	45.15 ± 9.1	42.93 ± 12.2	NS	44.36 ± 10.2	*0.029*
BMI (kg/m^2^)	26.97 ± 4.5	26.37 ± 7.3	NS	26.8 ± 5.5	25.61 ± 2.8	25.55 ± 3.8	NS	25.59 ± 3.2	NS
Heavy Drinking Markers									
LTDH (unit year)	13.65 ± 9.12	12.87 ± 8.95	NS	13.40 ± 9.1	17.61 ± 10.1	11.36 ± 8.7	NS	15.24 ± 9.9	0.370
TD90 (unit number)	1108.54 ± 615.1	946.29 ± 685.1	NS	1059.9 ± 636.3	1035.58 ± 501.5	941.35 ± 419.3	NS	1003.1 ± 469.3	0.666
HDD90 (unit number)	69.02 ± 23.3	65.05 ± 20.4	NS	67.8 ± 22.4	66.95 ± 27.4	77.60 ± 15.0	NS	70.62 ± 24.1	0.583
AvgDD90 (unit number)	14.85 ± 7.4	13.22 ± 7.51	NS	14.36 ± 7.4	14.81 ± 5.5	11.70 ± 4.0	NS	13.74 ± 5.2	0.682
NDD90 (unit number)	74.06 ± 20.0	69.04 ± 20.3	NS	72.56 ± 20.1	68.95 ± 26.2	79.0 ± 13.4	NS	72.41 ± 22.8	0.975
NNDD90 (unit number)	15.78 ± 19.9	20.86 ± 20.4	NS	17.3 ± 20.1	20.95 ± 26.1	11.0 ± 13.4	NS	17.52 ± 22.8	0.963
Primary Nutrition Measures under Study and Liver Injury Markers									
SHL	9.42 ± 2.3	8.39 ± 2.2	NS	9.1 ± 2.3	21.85 ± 13.4	16.91 ± 3.75	NS	20.1 ± 11.1	NA
Folate	704.49 ± 251.1	766.25 ± 252.7	NS	724.7 ± 251.2	618.84 ± 244.2	747.12 ± 379.2	NS	656.85 ± 289.1	0.268
AST (IU/L)	64.76 ± 67.0	68.04 ± 82.9	NS	65.86 ± 72.3	96.10 ± 86.3	151.81 ± 139.3	NS	115.87 ± 109.2	0.004
ALT (IU/L)	53.66 ± 34.24	46.04 ± 58.9	NS	51.09 ± 43.9	73.45 ± 46.0	91.55 ± 78.1	NS	79.87 ± 58.7	0.003
AST: ALT Ratio	1.09 ± 0.65	1.41 ± 0.57	0.036	1.20 ± 0.64	1.28 ± 0.62	1.57 ± 0.61	NS	1.38 ± 0.62	NS
Proinflammatory and Gu-barrier Dysfunction Measures (Sub-set: *n* = 47)									
IL-1β (pg/mL)	0.52 ± 0.29	0.54 ± 0.62	NS	0.52 ± 0.39	0.49 ± 0.28	0.48 ± 0.45	NS	0.49 ± 0.33	NS
LPS (EU/mL)	0.097 ± 0.06	0.088 ± 0.053	NS	0.094 ± 0.057	0.099 ± 0.06	0.108 ± 0.07	NS	0.102 ± 0.062	NS
LBP (ng/mL)	1132.85 ± 1303.53	3393.75 ± 4808.71	*0.066*	1741.55 ± 2796.25	2365.15 ± 3159.15	984.15 ± 513.68	NS	2001.72 ± 2767.34	NS

GR.1: patients with normal serum homocysteine; GR.2: patients with high serum homocysteine level; LTDH: Lifetime Drinking History; TLFB: Timeline Followback (Total drinks in the past 90 days (TD90), Number of drinking days in the past 90 days (NDD90), Drinks per drinking day in the past 90 Days (DPD90), Average drinks per drinking day in the past 90 days (AvgDD90), and Heavy drinking days in the past 90 Days (HDD90)); SHL: serum homocysteine levels; AST: aspartate aminotransferase; ALT: alanine aminotransferase; IL1β: Interleukin-1 beta; LBP: lipopolysaccharide binding protein; LPS: lipopolysaccharide. Italicized *p*-value: not applicable for co-variable analysis since same age-group. NA: not applicable, NS: not significant.

## Data Availability

The data presented in this study are available on request from the corresponding author.
